# Synergistic strategies in photodynamic combination therapy for cancer: mechanisms, nanotechnology, and clinical translation

**DOI:** 10.3389/fonc.2025.1607259

**Published:** 2025-07-30

**Authors:** Daohong Kan, Rong Ding, Huchuan Yang, Yuming Jia, Kaijian Lei, Zhongming Wang, Wei Zhang, Chaokun Yang, Zongjunlin Liu, Fang Xie

**Affiliations:** ^1^ Department of Burn and Plastic Surgery, The Second People’s Hospital of Yibin, Yibin, Sichuan, China; ^2^ Department of Clinical Medicine, Xinjiang Medical University, Urumqi, China; ^3^ Department of Oncology, The Second People’s Hospital of Yibin, Yibin, Sichuan, China; ^4^ Department of Internal Medicine, Gongxian Hospital of Traditional Chinese Medicine, Yibin, Sichuan, China; ^5^ Department of Rehabilitation, Gongxian Hospital of Traditional Chinese Medicine, Yibin, Sichuan, China; ^6^ Department of Thoracic Surgery, The Second People’s Hospital of Yibin, Yibin, Sichuan, China; ^7^ Department of Dermatology, The Affiliated Hospital of Southwest Medical University, Luzhou, Sichuan, China

**Keywords:** malignant tumor, combination therapy, curative effect, mechanism, imaging-guided

## Abstract

Photodynamic therapy (PDT), a non-invasive and highly selective method for cancer treatment, has gained increasing attention due to its unique ability to activate a photosensitizer with near-infrared laser irradiation, generating reactive oxygen species (ROS) and inducing cytotoxic effects on tumors. However, PDT faces challenges such as the shallow penetration depth of the laser impacting treatment efficacy and the variability in ROS yield depending on various factors. Recent advancements in nanotechnology have paved the way for solutions, showing promising results in addressing these limitations. Therefore, there is rising interest in utilizing PDT in combination with other therapeutic modalities to enhance its anti-tumor efficacy. This review aims to compile relevant basic experiments and clinical studies on the principles, mechanisms, and various combination therapies of PDT, including with photothermal therapy, radiotherapy, chemotherapy, targeted therapy, and immunotherapy. The findings from these studies consistently confirm that photodynamic combination therapy achieves a higher therapeutic index with lower side effects compared to the use of these modalities individually. The demonstrated synergistic effects and enhanced therapeutic outcomes in various studies underscore the need for additional research and development in this direction.

## 1 Introduction

In recent years, photodynamic therapy (PDT) has gained widespread recognition as a non-invasive and safe local treatment for tumors. Photosensitizers (PS) exhibit a distinct affinity for tumor tissues over normal tissues, resulting in significantly higher concentrations within the former. When the tumor target area is irradiated with a specific wavelength of laser light, the photosensitized tissue generates singlet oxygen and free radicals, damaging cells and inducing tumor cell death. Currently, PDT is widely used in the treatment of oropharyngeal cancer, esophageal cancer, and skin cancer (1). However, limitations such as low ROS yield, insufficient laser penetration, and the lack of targeting specificity and autotoxicity of photosensitizers restrict the clinical application of PDT in oncology (2). To enhance its efficacy, researchers have increasingly integrated PDT with various traditional cancer therapies, including photothermal therapy, chemotherapy, radiotherapy, targeted therapy, and immunotherapy (3). This article aims to elucidate the underlying mechanisms of PDT and highlight recent advancements in both basic and clinical research, particularly focusing on the synergistic effects of PDT in combination with other therapeutic modalities for tumor treatment.

## 2 Principles of photodynamic therapy

Photodynamic therapy (PDT) involves three main components: a photosensitizer, a laser with an appropriate wavelength, and oxygen dissolved in the cell. The PDT response is mediated through two primary mechanisms, both dependent on intracellular oxygen molecules (4). The initial phase involves the administration of a photosensitizer into the cell, followed by irradiation with a laser matching the photosensitizer’s absorption spectrum. This interaction activates the photosensitizer, generating reactive oxygen species (ROS) that cause cell damage and death. The photosensitizer transitions from the ground singlet state (S^°^) to the excited singlet state (S^1^) upon absorbing photon energy, with some energy emitted as fluorescence and the remainder directing the photosensitizer to the excited triplet state (T^1^), the most therapeutically active form. In the second phase, the excited triplet state facilitates ROS generation through two pathways: the Type I pathway involves electron transfer reactions generating free radicals and radical ions, while the Type II pathway involves energy transfer to molecular oxygen (^3^O_2_), producing highly reactive singlet oxygen (^1^O_2_). Singlet oxygen is highly oxidizing, causing photodamage to proteins, lipids, and other molecules, leading to cell death via apoptosis, necrosis, or autophagy, depending on the photosensitizer’s intracellular location (5–7).

**Figure 1:**
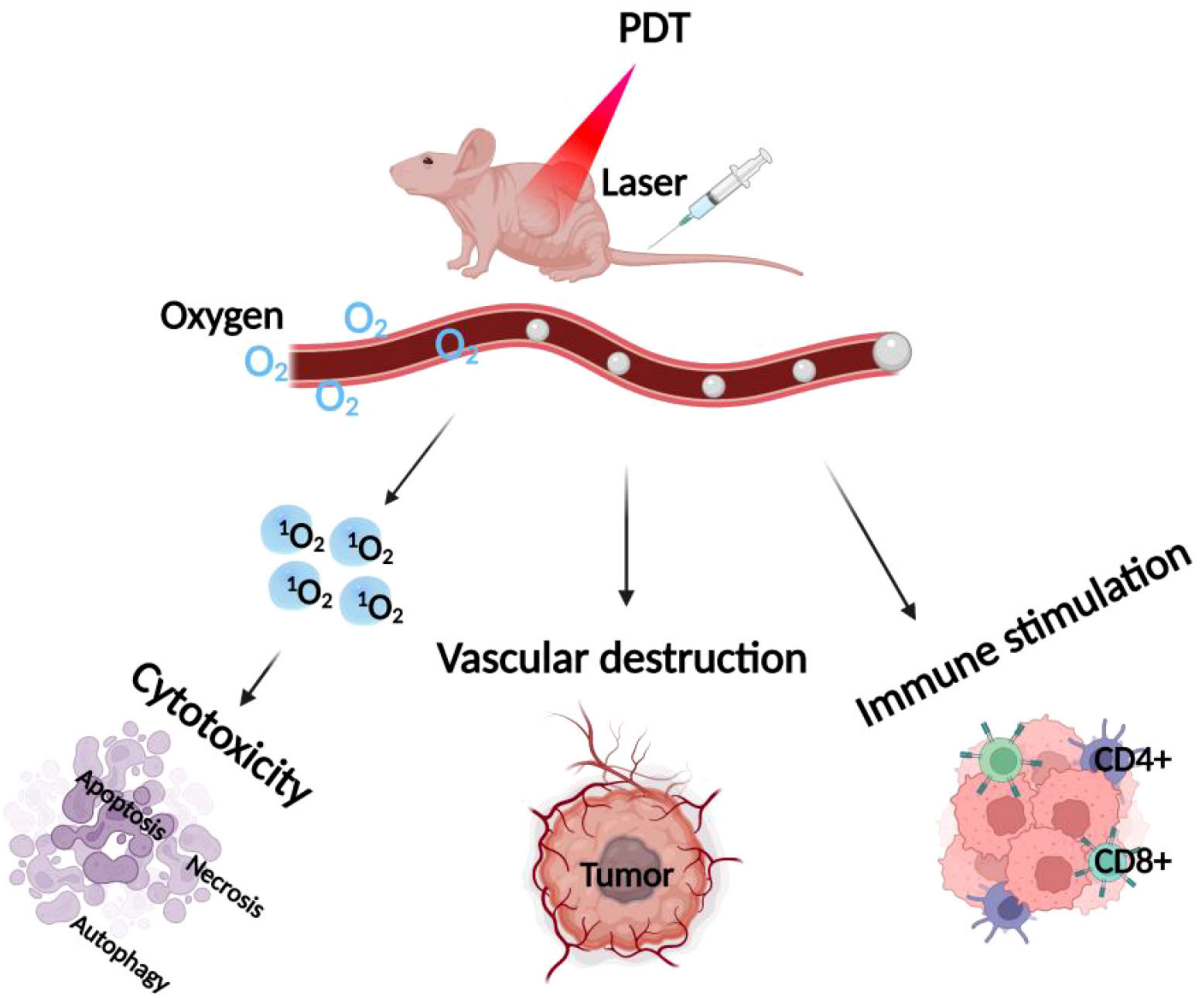
Mechanism of PDT. The diagram illustrates the primary mechanisms of PDT, which include: 1) direct cytotoxicity to cancer cells, 2) destruction of tumor blood vessels, and 3) stimulation of an autoimmune response.

The anti-tumor effects of PDT are mediated through three primary actions: (1) direct cytotoxicity to cancer cells, (2) destruction of tumor vasculature, and (3) stimulation of an autoimmune response (4). Direct chemical damage to tumor cells through ^1^O_2_ produced during the PDT reaction induces apoptosis, necrosis, and autophagic responses (8). Cancer cells that evade direct photocytotoxic effects may still suffer damage due to PDT’s impact on tumor vasculature. ROS damage to vascular endothelial cells activates coagulation, platelet aggregation, and thrombus formation, leading to vascular occlusion and persistent hypoxia, ultimately causing cell death (9, 10). Additionally, PDT induces a systemic anti-tumor immune response by destroying tumor structures, stimulating direct interactions between tumor immune cells and cancer cells. The destruction of tumor tissue triggers a strong inflammatory response and leukocyte infiltration, further exacerbating tumor damage (11, 12). Photodamage of the vessel wall attracts neutrophils and macrophages. Neutrophil degranulation as well as the release of lysosomal enzymes and chemokines promote the destruction of tumor tissue, exacerbating the destruction triggered by early photodamage, as shown in **Figure 1**.

## 3 Classification of photosensitizers and their characteristics

PDT has been used clinically or preclinically with hundreds of photosensitizers, such as porphyrins, dihydroporphyrinols, and phthalocyanine derivatives, all sharing a common tetrapyrrole structure. This macrocyclic structure facilitates effective light absorption and the generation of oxygen in a stable, linear state. Photosensitizers are commonly classified into three generations, with the first generation primarily consisting of hematoporphyrin derivatives (HpD), such as dihaematoporphyrin ether (DHE) and Photofrin, which have been approved for clinical use and are characterized by their long retention time in the body and a maximum excitation wavelength at 630nm. Despite its widespread use in PDT, the formulation exhibits specific limitations in clinical applications: 1) low chemical purity (it consists of more than 60 molecules); 2) poor tissue penetration due to maximal absorption at the relatively short wavelength of 630 nm; 3) a prolonged half-life of the photosensitizer, hotosensitizer and its high accumulation in normal organs, which produces corresponding side effects, for example, high accumulation of the photosensitizer in the skin, the skin is highly affected by prolonged exposure to the photosensitizer. Highly accumulates in the skin, and the skin becomes photosensitized due to prolonged exposure to PDT (13, 14).

Second-generation photosensitizers are primarily synthesized from hematoporphyrin derivatives. 5-Aminolevulinic acid (ALA), serving as a crucial precursor to protoporphyrin IX, is a prodrug that transforms into an active photosensitizer only upon conversion to protoporphyrin; hence, ALA and its esters can be administered topically or orally for numerous clinical purposes. Second-generation photosensitizers are synthesized from hematoporphyrin derivatives, with 5-aminolevulinic acid (ALA) being a key precursor to protoporphyrin IX. These photosensitizers offer enhanced chemical purity, single-linear oxygen release, and improved tissue penetration due to peak absorption in the 650–800 nm range. However, their low water solubility limits intravesical administration (15, 16).

Third-generation photosensitizers are designed for higher tumor tissue affinity and reduced damage to surrounding healthy tissues. Challenges remain in formulating drugs for parenteral delivery. To improve bioavailability and tumor selectivity, first- and second-generation photosensitizers have been modified with monoclonal antibodies against cancer cell-specific antigens and tumor surface markers, enhancing accumulation at tumor lesions and reducing drug dosage while maintaining therapeutic efficacy, as shown in **Table 1** (17–19).

**Table 1:** Classification and properties of photosensitizer generations.

Category	The first generation	The second generation	The third generation
Composition	Hematoporphyrin derivative (Hpd)	Modification of Hpd	PSs modified with bio-molecules or targeting agents.
Advantages	Clear efficacy	Stable structure, eliminate fast	Specificity, Efficacy
Disadvantages	Poor specificity, Skin phototoxicity	Low solubility, Low selectivity	Insufficient data, Limited clinical application
Representatives	Photofrin*	ALA*,Ce6,TPPS4,mTHPC*, (Np-(o-OH)H2TPP	Ac-L-PheLAOMe, P-Glu4 Or Gal4

(* represents FDA approved).

Notwithstanding the pledge and heartening results exhibited by PDT in the management of solid growths in preclinical trials, merely a minuscule proportion of nanomedicine-based methods have been effectively transferred into clinics. Obstacles relating to safety, efficacy, scalability, regulatory concerns and the lack of similarity between preclinical models and real tumors prevent targeted nanoplatforms from reaching clinical trials (1, 20).

In clinical practice, the following conditions are commonly treated with PDT: precancerous keratosis, skin lesions and some non-melanoma skin cancers (21). Furthermore, a number of solid tumor types, including esophageal, lung and prostate cancers, have been identified as suitable candidates for PDT in certain patients. There is a lot of evidence from clinical trials supporting the use of PDT as a treatment option for many other tumor types. These include cancers of the breast, head and neck, bile duct, bladder, pancreas, cervix, brain and other organs. Photofrin was the first PDT drug to be approved by the Food and Drug Administration (FDA) for different types of cancer. Porfimer sodium, a pure part of HpD, is the most commonly used photosensitizer for PDT of non-cutaneous solid tumors. In 1993, the granting of regulatory approval for porfimer sodium for the use of PDT in the treatment of bladder cancer in Canada was followed by FDA approval in 1995 for the palliation of symptoms in patients with obstructing esophageal cancer and in 1998 for non-small-cell lung cancer (NSCLC) indications (22). HpD was granted approval in China for oncological indications in 2001 (23). In 2003, this agent was also approved by the FDA for PDT of precancerous high-grade dysplasia in patients with Barrett esophagus.

Even though these approvals have been given, PDT is not used very much to treat solid tumor. Increased safety and efficacy of PDT, alongside broader indications and greater physician familiarity with the technique, are key to expanding its use in treating solid tumors. This can be achieved through technological advances.

## 4 Molecular mechanisms associated with photodynamic therapy

In PDT, an imbalance between ROS production and cellular repair leads to oxidative stress, triggering various cell death mechanisms. These mechanisms include apoptosis, necrosis, and autophagy, with the specific pathway depending on treatment conditions. High light damage typically induces necrosis, while moderate damage induces apoptosis, and mild damage triggers adaptive autophagy (24–26).

### 4.1 Apoptosis

Cellular apoptosis is commonly categorized into two primary pathways: the intrinsic pathway, which involves the mitochondrial release of apoptotic factors, and the extrinsic pathway, which is initiated by death receptors on the cell surface. The initial pathway is predominantly initiated by changes in mitochondrial outer membrane permeability or external stimuli, resulting in the activation of effector caspases 3 and 7 (Caspase-3/Caspase-7). However, when alterations in the extracellular environment are detected by membrane receptors in the cytoplasm, the extrinsic pathway is initiated. The extrinsic apoptotic pathway will be initiated, which is specifically associated with the involvement of Caspase-8. This pathway is characterized by the activation of Caspase-8, which subsequently triggers a cascade of caspase activations, ultimately leading to apoptosis. In both apoptotic pathways, the cytosolic membrane integrity is preserved *in vivo*, and immune cells such as macrophages efficiently phagocytose dying cells and apoptotic bodies. Whereas *in vitro*, membrane disruption occurs, which is often referred to as secondary necrosis (27).

Intrinsic apoptosis is the most common pathway of apoptotic cell death during photodynamic therapy. The BCL-2 family of proteins, which includes both pro-apoptotic and anti-apoptotic members, plays a crucial role in regulating cellular apoptosis. These proteins are categorized based on their BCL-2 homology domains (BH), with the anti-apoptotic proteins such as Bcl-2, Bcl-xL, Mcl-1, and Bcl-w, and the pro-apoptotic proteins including Bax, Bak, Bok, Bcl-xS, Bid, Bad, and Egl-1. The balance between these proteins, particularly the Bcl-2/Bax ratio, is a critical determinant in the initiation of apoptosis, with Bax forming homodimers to induce cell death and Bcl-2 forming heterodimers with Bax to inhibit apoptosis. The former have all four BH domains (BH1, BH2, BH3 and BH4) and include BCL-2, BCL-XL, MCL-1, BCLL2 and BFL-1 proteins. The latter include proteins with BH1, BH2, and BH3 domains, including BAX, BAK1, and other pro-apoptotic BCL-2 family members, and BH3 proteins containing only the BH3 domain (e.g., BAD, BID, PUMA, and BIM proteins) (28).

Intrinsic apoptosis may be caused by direct damage to mitochondria or indirectly stimulated by signaling pathways activated as a result of damage inflicted on other cellular structures. Sustained homeostatic disruption/damage to alternative mitochondrial sites (e.g. endoplasmic reticulum, ER) will induce activation of BH3 proteins (BID, BIM, PUMA, and NOXA proteins), which in turn activates BAX and BAK1 proteins and creates a pore in the mitochondrial outer membrane. This step of altered mitochondrial membrane permeability is irreversible and triggers the dissipation of mitochondrial membrane potential (Δψm) and the release of mitochondrial proteins into the cytoplasm (e.g., SMAC and cytochrome c).SMAC binds to members of the inhibitor of apoptosis (IPA) family of proteins including XIAP proteins, which inhibit caspase activity. Cytochrome c binds to APAF1, pro Caspases-9, to form apoptotic vesicles, which trigger the activation of Caspases-9, which then mediates the protein hydrolysis of effectors (Caspases-3) and executors (Caspases-7). The former cleaves different cellular substrates, leading to morphological and biochemical changes during apoptosis. This includes Caspases-3-induced phosphatidylserine (Phosphatidylserine) exposure and DNA fragmentation. Activated BAX and BAK also permeabilize the ER membrane (especially in the context of ER stress), which mediates the release of ER chaperone (Molecular Chaperone) and Ca2+ into the cytoplasm. The latter is taken up by mitochondria and contributes to their membrane permeabilization. Mitochondrial membrane permeability may be impaired by anti-apoptotic members of the BCL-2 family (BCL2, BCL-XL, MCL-1, BCLeW, BFL-1 proteins), which are commonly associated with the mitochondrial outer and ER membranes. They inhibit apoptosis by directly interfering with pro-apoptotic members of the BCL-2 family (i.e., BAX and BAK proteins), by preventing their oligomerization and pore-forming activity through physical chelation at the mitochondrial outer membrane, and indirectly by inhibiting activators of BH3 proteins (8, 29, 30). In addition, it has been reported that the levels of BCL-2 family members are altered in PDT treatments mediated by different photosensitizers, and an increase in the ratio of pro-apoptotic proteins to anti-apoptotic proteins (BAX/BCL-2) is usually observed. Accumulation of photosensitizers in mitochondria has been associated with the disruption of anti-apoptotic proteins such as BCL-2 (28, 31, 32).

### 4.2 Necrosis

Necrotic or accidental cell death is a rapid and uncontrolled form of cell death caused by intense physical or chemical damage. The process leads primarily to the disruption of ion pumps in the plasma membrane, which is accompanied by an increase in membrane permeability, ultimately leading to water and ion influx, swelling of cells and organelles, cytoplasmic vacuolization, and chromatin clumping. In contrast to regulatory cell death, necrosis is considered to be a passive process that does not require protein synthesis and the regulation of energy or signaling pathways. Studies have shown that mitochondria, endoplasmic reticulum, and lysosomes play important roles in regulating necrosis. The breakdown of lysosomes causes the release of a number of proteases into the cytoplasm, such as tissue protease (Cathepsin) and calpain. In addition, Ca^2+^ is elevated in the cytoplasm, which is essential for activating the translocation of calpain and phospholipase A2 (PLA2) to the cell membrane, which mediates the disruption of the cell membrane. The rupture of the plasma membrane releases all cellular contents, leading to intense inflammation (33–35).

In recent years, it has been widely recognized by researchers that PDT by applying high photosensitizer concentrations and/or high light doses usually kills cells by inducing necrosis. In addition, it has been shown that the onset of necrosis is also related to the distribution of photosensitizers on the plasma membrane, which mediates the loss of membrane integrity and ATP depletion upon photoactivation. Short incubation times favored photosensitizer accumulation at the plasma membrane, while longer incubation times allowed photosensitizers to accumulate in different organelles. Cells incubated by researchers using the photosensitizer Photofrin for short periods (e.g., 1–3 h) showed accumulation of photosensitizer primarily in the plasma membrane, whereas longer incubation times (e.g., 24 h) resulted in preferential accumulation of photosensitizer in the Golgi apparatus (GA). Interestingly, upon photoactivation, the former promoted cell death through necrosis, whereas apoptosis was observed when the GA was the target organelle, suggesting that plasma membrane accumulation is a preferential target for necrosis (30, 36, 37).

### 4.3 Autophagy

Autophagy is a catabolic process that functions as follows: a) a mechanism that contributes to survival by removing damaged cellular material (adaptive autophagy); and b) a mechanism of death (autophagy-dependent cell death). Each of these relies on the formation of a double-membrane vesicle that engulfs damaged material isolated from the cytoplasm (autophagosome) and then fuses with lysosomes, leading to digestion of the autophagosomal body membrane and the formation of a single-membrane vesicle (autolysosome). It is within the autolysosome that autolytic material is degraded by lysosomal hydrolases, and degradation removes both damaged material (e.g., unfolded proteins, damaged organelles, or microorganisms) and reuses the recovered nutrients for normal cellular processes. Autophagosome formation is a highly complex and regulated process involving many proteins and signaling pathways, such as a) ULK1 complex; b) BECN1 complex; and c) autophagy-related genes (ATG). In addition, the LC3 protein is one of the key proteins in autophagosome formation and its substance selection. It is normally redistributed from a diffuse cytoplasmic pattern (LC3-I) to so-called autophagic sites (LC3-II), a process that requires a series of ubiquitin-like reactions to mediate the binding of LC3-I to the phosphatidylethanolamine lipids (PE) of the autophagic vesicles and the production of LC3-II. Indeed, the lipolytic action of LC3 and the detection of LC3-positive sites are the most common markers for the recognition of autophagy (30, 38, 39).

The function of autophagy as a cell survival or death mechanism during photodynamic therapy depends on the degree of induced photodamage. After autophagy activation, death may occur directly as a result of the action of the autophagy mechanism (autophagy-dependent cell death) or activation of regulated cell death (usually apoptosis) (40). Numerous findings have shown that inhibition of autophagy inhibitors or autophagy genes usually increases ROS-mediated phototoxic damage, thus suggesting that autophagy is a survival mechanism rather than a cell death pathway. For example, PDT mediated by the photosensitizers CPO and chrysin (both ER-targeted) both showed apoptosis induction with signs of autophagy (e.g., double-membrane autophagosomes, LC3 lipidation, and cytoplasmic vacuolization). In both studies, inhibition of autophagy by PI3K inhibitors (e.g., Warman’s penicillin) or silencing of autophagy genes (e.g., ATG5) resulted in an increase in cell death, which reveals that autophagy has a protective role against phototoxic damage to cells. However, this pro-survival effect can only occur at a certain level of photodamage threshold (occurring only under low light conditions). However, PDT-triggered autophagy-dependent cell death occurred mainly in apoptosis-inhibited cells. Another study found that cell death without signs of apoptosis but with LC3 lipidation and cytoplasmic vacuolization (signs of autophagy) was observed when CPO-PDT was performed in BAX-deficient cells and was significantly inhibited by PI3K inhibitors (41, 42).

## 5 Combined photodynamic therapy strategies

**Figure 2:**
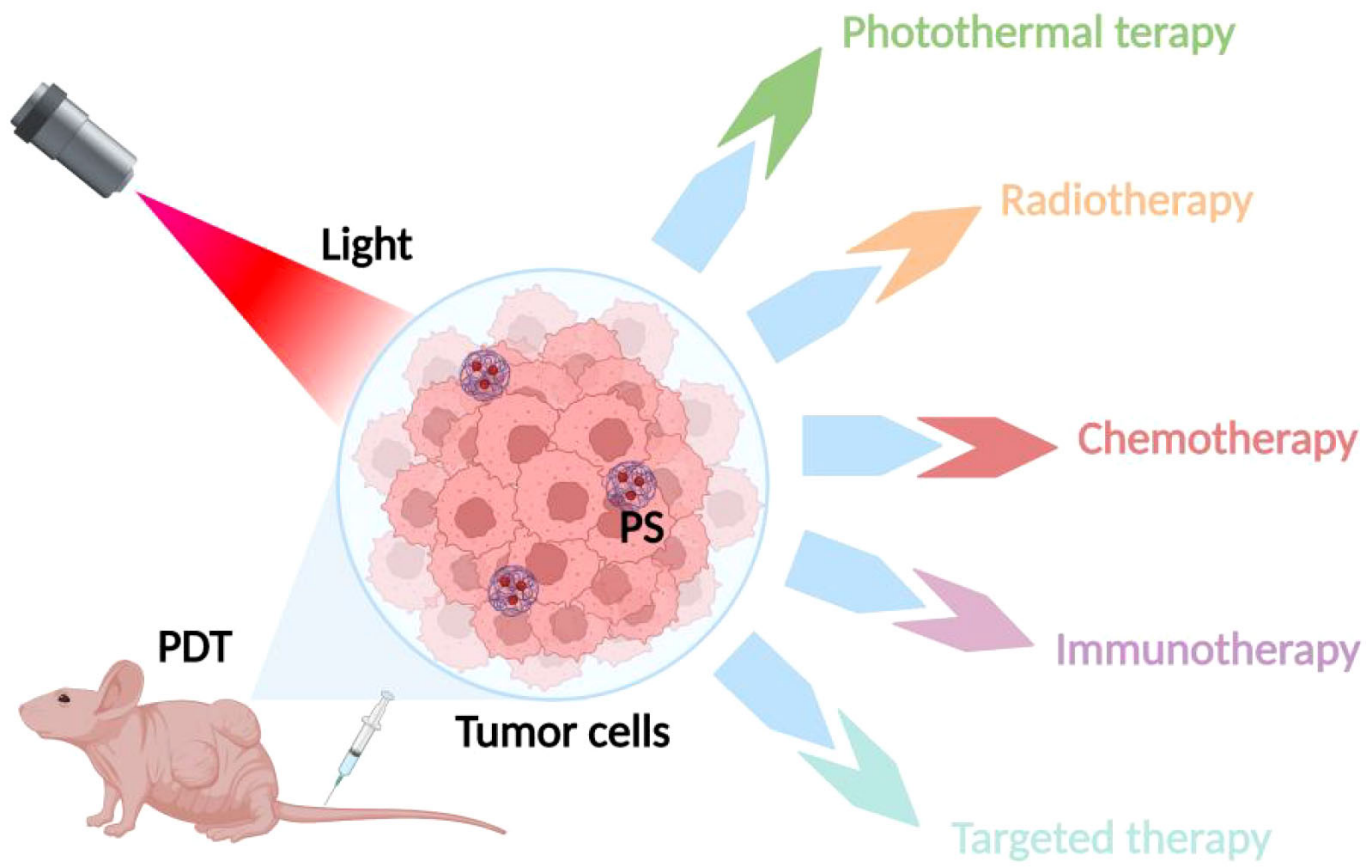
Strategies of combined PDT therapies. The various therapeutic strategies combined with PDT, highlighting combinations with photothermal therapy, radiotherapy, chemotherapy, targeted therapy, and immunotherapy.

The development of photosensitizers and phototherapeutic devices has come a long way in past research, but key challenges faced during photodynamic therapy still limit its use in antitumor therapy. Photosensitizers have limited selectivity for tumor tissues, and for surrounding nonmalignant tissues, thus necessitating the use of large doses to ensure efficacy. Consequently, the accumulation of photosensitizers in tissues and organs adjacent to tumors and stray light beyond the volume of the treated tumor can lead to collateral damage (43). In addition, PDT requires an exogenous light source for excitation, and the limited depth of laser light at conventional wavelengths through biological tissue means that PDT is usually ineffective for deep tumors. Moreover, PDT has poor efficacy against hypoxic tumors and its therapeutic effect is oxygen-dependent (44). It has been found that once the PDT response is activated by light, it rapidly induces severe local hypoxia by depleting tissue oxygen disrupting blood flow within the tumor, and ultimately stopping ^1^O_2_ production. It has also been found that most metastatic tumors develop areas of severe hypoxia, and in addition, PDT action causes acute hypoxia, which reduces cellular oxygen reserves and ultimately decreases the efficacy of PDT therapy (45). In addition, in order to minimize the potential skin toxicity caused by the excitation of residual photosensitizers, patients need to rest in a dark environment for a considerable period of time after PDT, which is inconvenient for patient treatment (46). Therefore, to improve the therapeutic efficacy of photodynamic therapy, more and more researchers have tried to combine PDT with other traditional antitumor therapies (including photothermal therapy, radiotherapy, chemotherapy, targeted therapy, and immunotherapy), to take full advantage of their respective strengths and make up for each other to produce synergistic therapeutic effects, to improve therapeutic efficacy and reduce the adverse effects, as shown in **Figure 2**. In recent years, nanotechnology has been widely used in the study of photodynamic combination therapy, and nanocarriers play an important role in integrating different therapies. The integration of PDT with other advanced technologies, such as nanotechnology, further boosts its potential in treating drug-resistant tumors. The use of nanoparticles as carriers for photosensitizers or as energy donors can enhance the delivery and efficacy of PDT. Additionally, combining PDT with other therapies like radiotherapy and immunotherapy could provide a comprehensive approach to tackling cancer, especially in cases where tumors have developed resistance to standard treatments. These combinatorial strategies highlight the innovative potential of PDT in overcoming drug resistance and improving patient outcomes in cancer therapy (47). Next, we will make a summary of the related research and progress of photodynamic combination therapy, hoping to better guide the clinical application of photodynamic combination therapy.

### 5.1 Photodynamic therapy combined with photothermal therapy strategies

In photothermal therapy (PTT), photothermal agents (PAs) enhance the heat of cells and tissues in a localized area. When PAs are irradiated by a laser of a specific wavelength, they absorb energy from photons and transition from the ground singlet state S^°^ to the excited singlet state S^1^. Subsequently, the electronic excitation undergoes vibrational relaxation (a non-radiative form of decay), which mediates the return to the ground state through collisions between the excited PA and its surrounding molecules. As a result, the increase in kinetic energy causes the surrounding microenvironment to heat up. When the temperature of the tissue rises to 41°C, a heat shock response occurs, which in turn causes rapid changes in a range of gene expression patterns, including the production of heat-excited proteins to mitigate the effects of the initial heat damage. When the temperature rises to 42°C, irreversible tissue damage will occur. Heating tissue to temperatures of 42-46°C for 10 minutes results in irreversible cell necrosis. At 46-52°C, cells die rapidly due to microvascular thrombosis and local ischemia. At tissue temperatures greater than 60°C, which can usually be achieved by PTT, cell death occurs almost instantaneously due to denaturation of proteins and DNA and disruption of the plasma membrane (1, 48).

**Figure 3:**
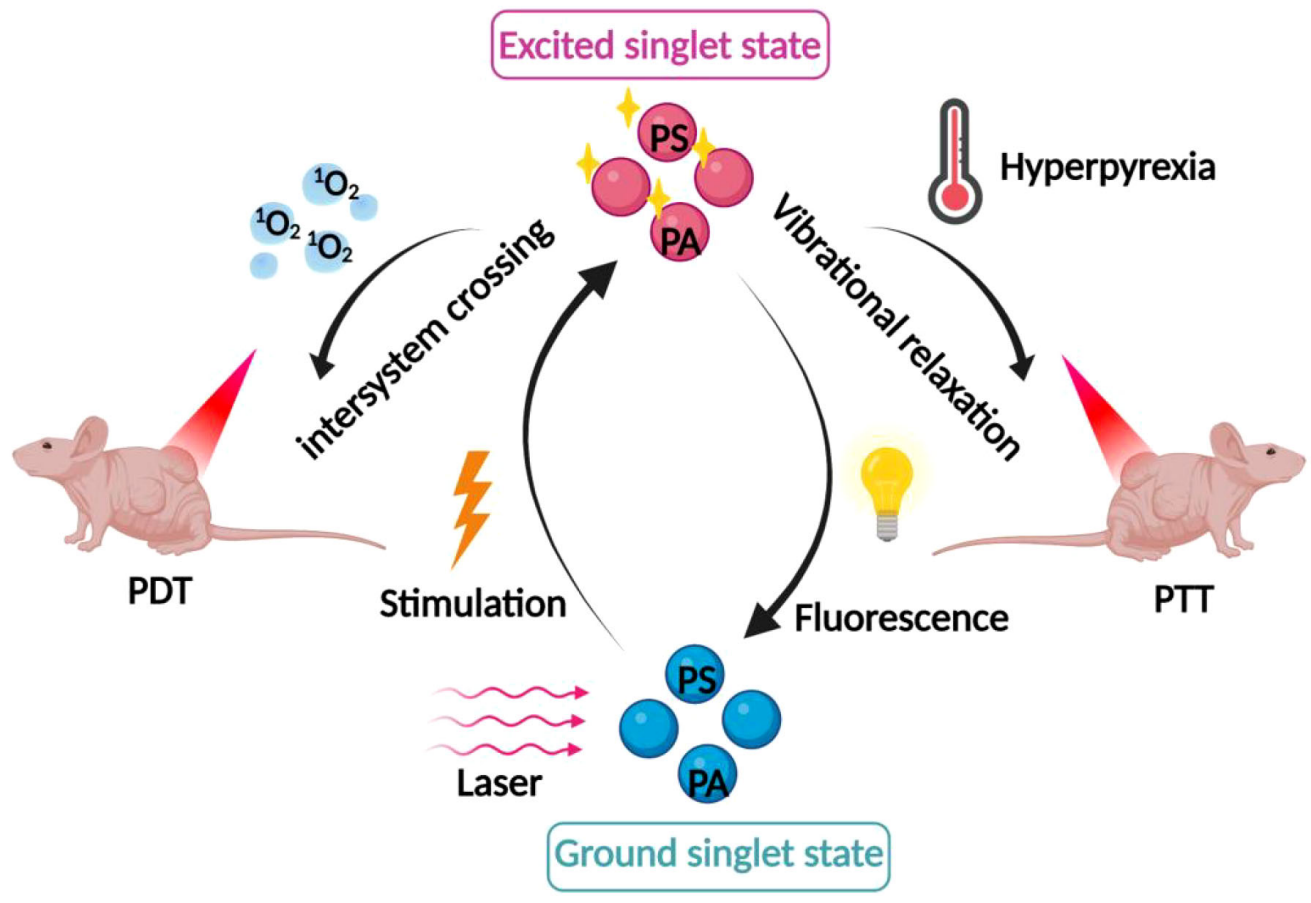
Mechanism of combined PDT and photothermal therapy. The synergistic effects of combining PDT with photothermal therapy, where the thermal effect enhances intracellular delivery of photosensitizers, improves local blood supply, and increases oxygen content within the tumor.

In recent years, researchers have proposed a novel therapeutic model combining PDT and PTT therapies, which shows better anti-tumor effects through the synergistic effect of PDT and PTT, as shown in **Figure 3**. It was found that the thermal effect of PTT could increase the intracellular delivery of photosensitizers improve the local blood supply within the tumor and increase the oxygen content in the tumor to promote the production of ROS, resulting in higher PDT efficacy. At the same time, ROS generated during PDT can in turn destroy heat shock proteins and subsequently protect normal cells from PTT. A better anti-tumor effect will be obtained through the synergistic effect mediated by ROS generated by PDT and PTT-induced hyperthermia (49, 50). Wang et al. coupled gold nanoparticles with the photosensitizer dihydroporphyrinol e6 (Ce6) via amide bonds, and then modified the PEG on the surface, to prepare a novel nano-PDT/PTT combined therapeutic system, AuNPs-PEG-Ce6. The experimental results showed that AuNPs-PEG-Ce6 had a higher ROS generation rate and stronger PDT and PTT effects than free AuNPs-PEG and Ce6, with stronger anti-tumor effects. Another study on gold nanoparticles also found that gold nanoparticles have not only good photothermal effects, but also strong photodynamic effects, and the mechanism may be attributed to the generation of hot electrons by surface plasmon resonance relaxation (SPR) under the excitation of near-infrared light (NIR), which subsequently sensitizes the interfacial oxygen through both electron transfer and energy transfer modes, thus inducing the generation of ROS. The generated ROS can trigger the mitochondria to be more active, and the mitochondria can be more active. The generated ROS can trigger mitochondrial damage, which in turn induces the up-regulation of apoptosis-related proteins, ultimately leading to apoptosis of tumor cells. In addition, since tumor cells in weakly acidic microenvironments inherently contain H_2_O_2_, an increase in its concentration can induce malignant transformation of the cells (51). The active intermediates of gold nanoparticles during PTT can also promote the decomposition of intracellular H_2_O_2_ to generate ROS, thus exerting phototoxic effects (52, 53). This lays a theoretical foundation for the application of gold nanoparticles in combined tumor photothermal photodynamic therapy. In addition, trivalent copper sulfide nanomaterials have been applied in cancer therapy due to their NIR-responsive properties. Hou et al. synthesized a novel nanomedicine PVP-Cu-Sb-S functionalized with poly(vinylpyrrolidone) (PVP). The results of the *in vitro* experiments showed that PVP-Cu-Sb-S exhibits high photothermal conversion efficiency (~53.16%) and induces a large amount of ROS production, showing an excellent PDT/PTT effect. Meanwhile, excellent tumor ablation effects were obtained in *in vivo* experimental hormonal mice without significant side effects (54). Indocyanine green (ICG) is known to be a photosensitizer for both PTT and PDT. Xia et al. proposed a promising strategy for PDT/PTT combination therapy by utilizing ICG chemically coupled with the photothermal converting agents, polydopamine (PDA) and tirapamine (TPZ), a hypoxia-activated prodrug. Under NIR laser irradiation, both ICG oxygen depletion and increased hypoxia at the tumor site activated TPZ to destroy DNA in the nucleus of tumor cells, while ROS generated by ICG synergized with PDA to enhance the efficiency of phototherapy. It was found that ICG-PDA-TPZ nanomedicine significantly improved tumor intracellular uptake, and at the same time showed strong PDT/PTT synergism in both *in vitro* and *in vivo* experiments, with excellent antitumor effects, and the drug itself had low toxicity, which is an effective strategy to improve the efficacy of phototherapy (55).

### 5.2 Photodynamic therapy combined with radiotherapy strategy

Radiation therapy (RT) has become one of the main means of treating malignant tumors, and about 70% of cancer patients need to undergo RT. The mechanism of action of RT is twofold: a) direct damage is mainly because rays can be absorbed by key cellular structures, including DNA, cell organelles, and cell membranes, which induces the production of free radicals, resulting in the DNA molecules to appear breaks and crossovers; b) indirect damage is mainly due to the ionization of rays with water-containing cytoplasmic solutes in tissues, which generates highly reactive free radicals and acts on biological macromolecules, leading to their stable and irreversible damage. These two effects are of equal importance during tumor regression after RT (56). However, it has been found that: the inherent properties of the tumor hypoxic microenvironment limit the efficacy of radiotherapy, as tumor cells are induced to develop radiation resistance in hypoxia. In addition, the self-repairing properties of DNA can weaken the tumor eradication ability of RT and even lead to cancer progression through DNA replication or random repair of broken DNA strands. In addition, non-uniform dose distribution of radiation sources is one of the reasons why RT treatment fails in clinical practice (57, 58).

**Figure 4:**
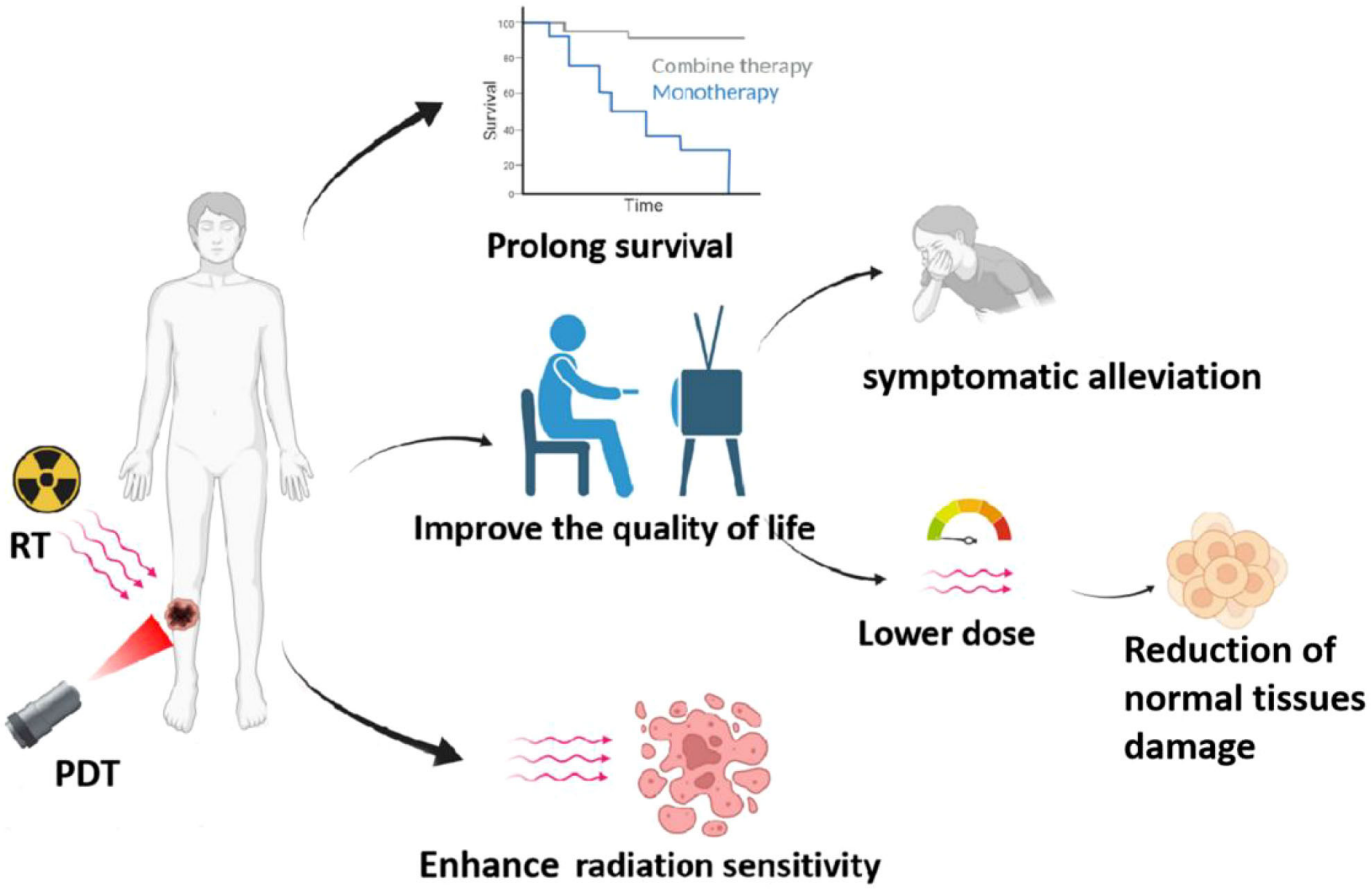
Benefits of PDT combined with radiotherapy, including: 1) prolonging patient survival, 2) improving sensitivity of radiotherapy, and 3) enhancing the quality of life for patients.

In recent years, to address the problem of “decreased sensitivity to radiotherapy in patients with intermediate and advanced tumors and the poor effect of single RT”, researchers have combined PDT with RT to improve the limitations of single therapy through a new combined treatment mode, as shown in **Figure 4**. The study found that radiotherapy is more effective for deep tumors due to the high tissue penetration ability of X-rays. However, for PDT, which relies on laser irradiation, the laser’s limited ability to penetrate tissues leads to its suboptimal efficacy for deep tumors. Therefore, RT combined with PDT can improve the poor therapeutic effect of single PDT on deep tumors. At the same time, both RT and PDT can induce the generation of ROS, so the combination treatment will obtain the anti-tumor synergistic enhancement effect. In addition, due to the low toxicity of PDT, the combination of PDT with RT can reduce the dose of RT and alleviate the toxicity of side effects while exerting synergistic effects. It has also been found that when PDT is combined with RT, PDT can first eliminate a portion of tumor cells, improve the blood supply in the tumor, and then reduce the proportion of hypoxic cells and enhance the sensitivity of RT (58, 59). Chen et al. reported nanodrugs (GRDs) formed by the coordination polymerization of gadolinium (Gd) with the photosensitizer Rose bengal (RB) for PDT/RT combination therapy of tumors. In ex vivo experiments, GRDs showed enhanced optical properties and photodynamic effects compared to free RB, with an enhancement of luminescence intensity by about 7.7-fold and an increase in ROS yield by about 1.9-fold; furthermore, GRDs showed better X-ray absorption and a 2-fold increase in r1 relaxation in MR imaging compared to gadopentetic acid (Gd-DTPA). More importantly, the study found that the PDT/RT combination significantly inhibited tumor growth compared to monotherapy (i.e., PDT or RT). This study provides a new avenue for Gd-based nanomedicines for MR imaging-guided PDT/RT combination therapy of cancer (60). Liu et al. applied a nano metallic organic skeleton (NMOF) composed of hafnium (Hf) and the photosensitizer, 4-carboxy phenyl porphyrin (TCPP), for PDT/RT combination therapy of tumors. Among the Hf-TCPP NMOFs, TCPP acts as a photosensitizer to exert an effective PDT effect; Hf has a strong X-ray attenuation ability and can be used as a radiosensitizer to enhance the efficacy of RT; and the polyethylene glycol (PEG) coating on the surface showed an effective tumor-nesting effect; especially in *in vivo* experiments in mice, the Hf-TCPP NMOFs significantly inhibited the tumor growth and the drug was efficiently cleared *in vivo*. The drug was effectively cleared *in vivo*, minimizing its possible long-term toxicity, which has a strong potential for clinical application (61). Antosh et al. designed a copper-cysteamine nanoparticle (pHLIP@Cu-Cy) modified by the PH-responsive targeting peptide pHLIP for tumor X-ray-induced PDT. Cu-Cy, as a novel photosensitizer, was activated by X-rays to produce a cellular-induced PDT. Cu-Cy, a novel photosensitizer, can generate cytotoxic ROS after activation by X-rays. pHLIP@Cu-Cy can bind to the tumor cells *in vivo* and perform PDT under the activation of X-rays, resulting in a significant reduction of tumor size. Due to the strong penetration ability of X-rays into deep tissues, pHLIP@Cu-Cy can be used for X-ray-induced PDT in deep tumors, and this novel strategy will surely improve the existing limitations of PDT and bring about better anticancer efficacy (62). In addition, the study by Sun et al. is dissimilar to the study above. It is well known that radionuclides with Cherenkov radiation (CR) can be used as an internal excitation source to activate photosensitizers for PDT of deep-seated tumors; however, the inefficiency of CR limits its therapeutic efficacy. Sun et al. developed a ^131^I-labeled zinc 4-carboxyphenoxyphthalocyanine (ZnPcC_4_)-conjugated Cr^3+^-doped zinc gallate (ZnGa_2_O_4_:Cr^3+^, ZGCs) nanoplatforms (^131^I-labeled ZnGa2O4:Cr^3+^, ZGCs) for PDT of deep-seated tumors (62). ZGCs) nanoplatform (^131^I-ZGCs-ZnPcC_4_) for tumor RT and X-ray-induced PDT. ^131^I can directly kill cancer cells not only by Cherenkov luminescence (CL), but also by activating persistent luminescence of ZGCs by ionizing radiation, and further by successive activation of the photosensitizer ZnPcC_4_ for PDT. In both *in vivo* and ex vivo experiments, ^131^I-ZGCs-ZnPcC_4_ showed excellent tumor inhibition. Combined with its self-activating PDT and RT effects, ^131^I-ZGCs-ZnPcC_4_ will greatly improve the efficacy of deep tumor therapy (63).

### 5.3 Photodynamic therapy combined with chemotherapy strategies

Chemotherapy is one of the most effective means of treating cancer, which is achieved through the use of chemotherapeutic drugs to kill cancer cells. Studies have shown that the anti-tumor mechanism of chemotherapeutic drugs usually involves: binding to the DNA of the cancer cells to inhibit the process of cell division, thereby curbing their DNA replication and ultimately leading to the death of the cancer cells. Although chemotherapeutic drugs can kill cancer cells to a certain extent, the lack of specificity of the drugs can cause serious toxic side effects throughout the body. In addition, chemotherapeutic drugs are prone to drug resistance, which greatly limits their clinical application (64).

**Figure 5:**
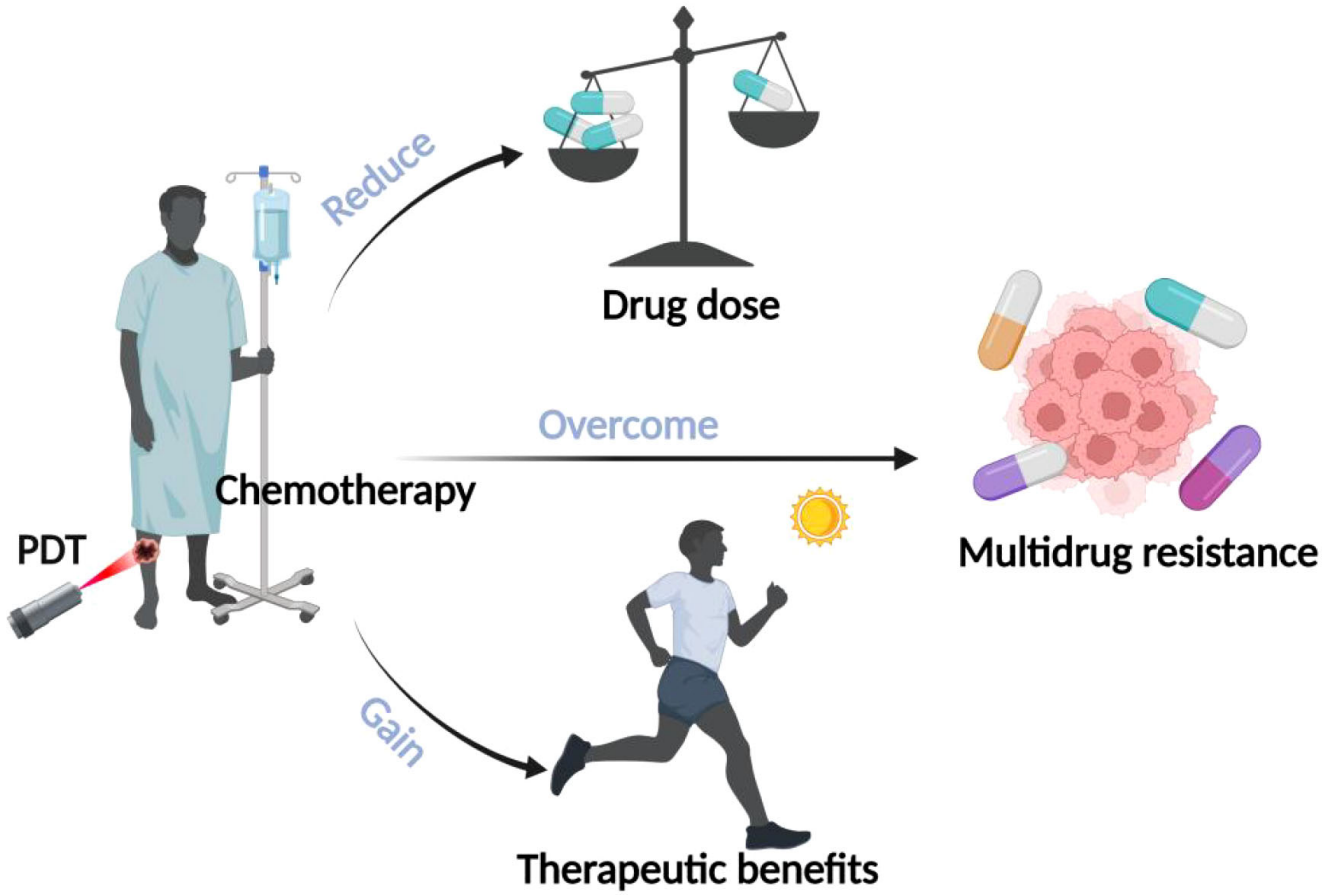
Advantages of combined PDT and chemotherapy. The illustration specifies the benefits of combining PDT with chemotherapy: 1) lowering the dosage of drugs, 2) overcoming multidrug resistance, and 3) gaining therapeutic benefits.

To overcome the toxic side effects and drug resistance of drugs to improve the efficacy of chemotherapy, more and more researchers have tried to combine PDT with chemotherapy to exert synergistic anti-tumor effects and reduce the dose of chemotherapeutic drugs, as shown in **Figure 5**. Casaba et al. found that low-dose treatment with adriamycin (ADM) before PDT resulted in a significant increase in the lipid peroxidation product malondialdehyde (LPO) LPO is an oxidative degradation product of lipids and is an indicator of cellular damage by reactive oxygen species. Researchers concluded that low-dose chemotherapy pretreatment can increase the sensitivity of cancer cells to ROS generated in the action of PDT, thus enhancing its killing effect on tumor cells (65). The study by Wang et al. constructed a novel 1O2-responsive nanocarrier, NOP-DOX@ BSA-FA, as a delivery system to unite the action of PDT and chemotherapy, while enabling the chemotherapeutic drug DOX can reach the tumor site more easily to effectively kill cancer cells, thus reducing the toxic side effects of chemotherapy (66). Husain et al. found that cisplatin-based chemotherapy combined with riboflavin-based PDT as a photosensitizer significantly reduced the genotoxicity of cisplatin in epidermal keratinocytes (67). In addition, it has been found that the mechanism of multidrug resistance (MDR) involving cell membrane efflux pumps is the main reason for the failure of chemotherapeutic treatment, and MDR is mainly related to the overexpression of P-glycoprotein on the surface of tumor cells. The ROS generated by photosensitizers during PDT can inhibit the function of drug efflux P-glycoprotein pumps in MDR cells, thus effectively reducing chemotherapeutic MDR. It can be seen that the combination of PDT with chemotherapy can overcome multidrug resistance generated during tumor treatment, thus improving the therapeutic efficacy (68). Therefore, more and more researchers have begun to develop novel drug delivery systems to improve the accumulation of chemotherapeutic drugs into the cytoplasm or nucleus by avoiding or reducing drug efflux. A study by Khdara et al. found that methylene blue-mediated PDT combined with the chemotherapeutic drug adriamycin (DOX) exhibited potent cytotoxicity against drug-resistant tumor cells due to the presence of drug-resistant cells after PDT therapy high concentrations of DOX, along with a decrease in P-glycoprotein expression and an increase in reactive oxygen species yield in tissues, which ultimately led to necrosis or apoptosis of drug-resistant tumor cells (69).

In addition, PDT in combination with chemotherapy is advantageous in improving the outcome of patients with advanced disease. Radical surgery is impractical for most patients with advanced cancer, and these patients are left with only palliative options to improve their quality of life and survival time. Wentrup et al. collected 68 patients with non-resectable cholangiocarcinoma of the hilar portion of the liver (NCC), who were treated with either PDT in combination with chemotherapy for PDT for hepatoportal bile duct carcinoma (PDT-C) or PDT alone for PDT for hepatoportal bile duct carcinoma (PDT-M). The results of the study found that the mean survival time was 374 days (n=35) in the PDT-M group and 520 days (n=33, p=0.021) in the PDT-C group, which indicated that the PDT combination therapy group had better efficacy than the treatment alone group (70). Kimura et al. selected 12 patients (8 males, 4 females) with advanced non-small cell lung cancer. These patients underwent PDT combined with chemotherapy to control localized lesions in the lumen. The results of the study found that the median bronchial official lumen stenosis rate before and after treatment was 60% and 15%, respectively (p<0.05). The data suggest that PDT combined with chemotherapy improves the quality of life and relieves bronchial obstruction in patients with advanced non-small cell lung cancer (71).

### 5.4 Photodynamic therapy combined with immunotherapy strategies

One of the newer options for cancer treatment is immunotherapy, which fights cancer cells by inducing a tumor-specific immune response in the body, either actively or passively. Since chemotherapy or radiotherapy is non-specific cytotoxic to any cell, including normal cells that are growing or dividing, immunotherapy overcomes this specificity problem by not producing cytotoxicity to normal cells lacking cancer antigens, sparing the body from injurious treatments (72, 73). In recent years, it has been found that local photodynamic therapy (PDT) is effective in inducing systemic anticancer immune responses through localized photodynamic therapy of tumors, and has good efficacy in tumors with occult foci or distant metastases. PDT has been reported to cause immunogenic cell death (ICD), which induces the release of damage-associated molecules to enhance tumor immunogenicity. Typically, the following four mechanisms underlie the way in which PDT can enhance immunotherapeutic responses (1): effective destruction of the treated tumor is permitted by ICD induced by PDT with contributions by local immune cells (2); antigens released during the process of ICD can act as an *in situ* vaccine, provided they are specific to the tumor; (3) the typically weak immunogenicity of native tumor antigens is boosted by damage-associated molecular patterns; and (4) The immune system is activated by pro-inflammatory cytokines being upregulated.

The effects mentioned can work together with those of immunotherapies. Some immunotherapies make the tumor more immunogenic, for example by using immunoadjuvants. Others reduce immunosuppression in the tumor environment, for example by using immune-checkpoint inhibitors (such as anti-PD-1, anti-PD-L1 and anti-CTLA4 antibodies). The result is an increase in the number of cytotoxic CD8^+^ T cells and effector memory T cells in the tumor (74–76).

For clarity, “cold” tumors are those with lower immunogenicity, immune cell infiltration, or lower T cell inflammatory profiles, which are resistant to immunotherapy, while “hot” tumors are those with increased T cell recruitment and are more responsive to immunotherapy. Examining the distinct responses and efficacy of PDT in these tumor types will provide a comprehensive understanding of its clinical application (77).

Photodynamic therapy (PDT) is a treatment modality that utilizes photosensitizing agents, light, and oxygen to induce cell death, primarily in cancer cells. The efficacy of PDT can vary significantly between “cold” and “hot” tumors, a distinction based on the level of immune cell infiltration within the tumor microenvironment (78). “Cold” tumors are characterized by low immune cell infiltration and are often resistant to conventional therapies, including PDT, due to their immunosuppressive nature. In contrast, “hot” tumors have a high degree of immune cell infiltration, making them more responsive to treatments like PDT.

The role of signaling pathways, such as the STAT3 pathway, is crucial in understanding the differential response of cold and hot tumors to PDT. STAT3 is known to play a significant role in maintaining the immunosuppressive environment of cold tumors by regulating the secretion of immunosuppressive molecules and the function of immunosuppressive cells. This regulation hinders the conversion of cold tumors into hot ones, thereby reducing the efficacy of PDT and other immunotherapies in cold tumors. Recent studies have highlighted the potential of targeting STAT3 to enhance the immune response and convert cold tumors into hot tumors, thereby improving the effectiveness of PDT and other therapeutic strategies (79).

By understanding the molecular mechanisms that differentiate cold from hot tumors, researchers can design new therapeutic strategies that aim to “heat” tumors. Such approaches could provide a significant advancement in the field of tumor immunotherapy, offering new hope for patients with traditionally hard-to-treat cold tumors.

**Figure 6:**
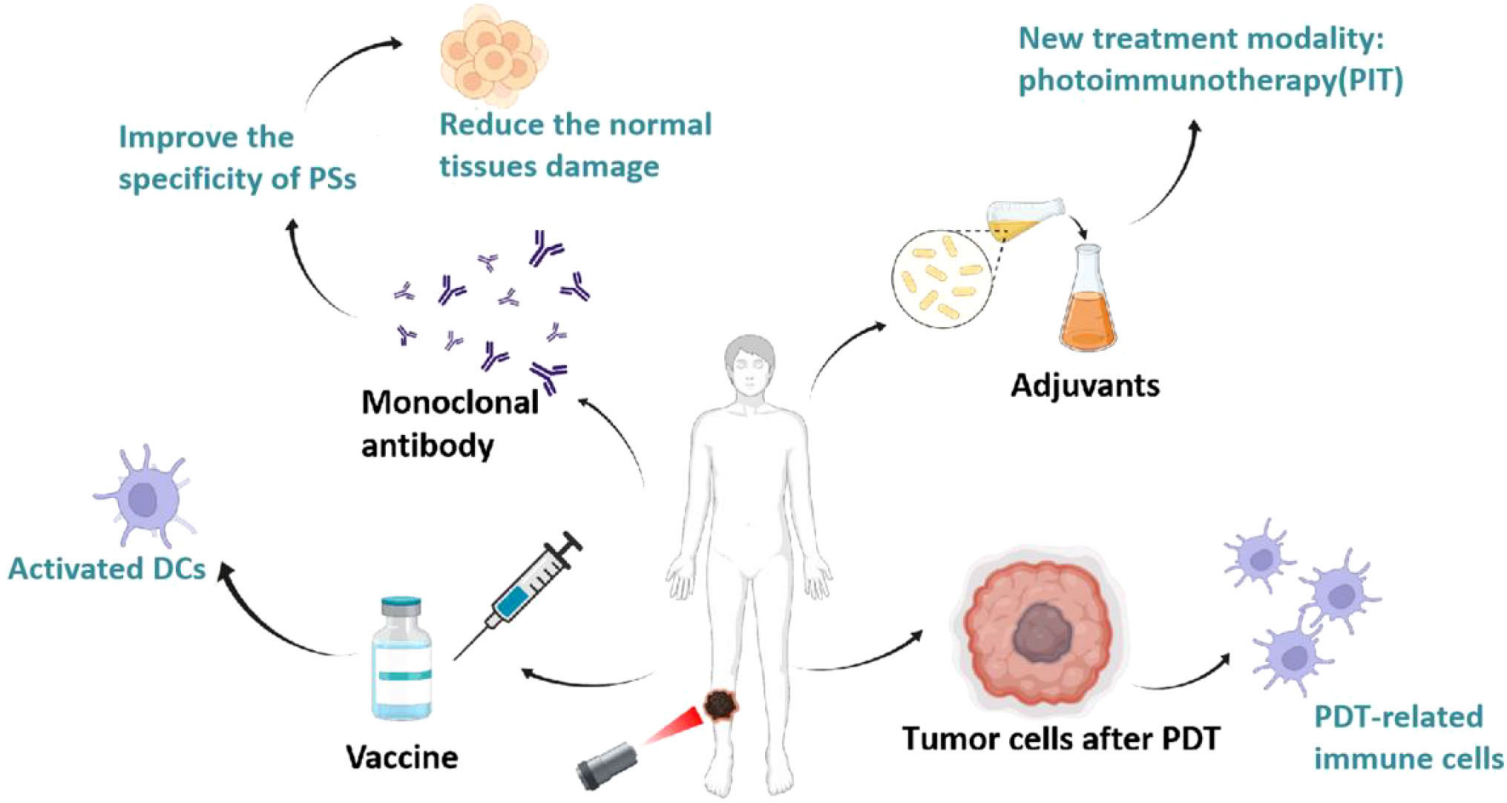
Advantages of combined PDT and immunotherapy. The synergistic effects of combining PDT with immunotherapy, including: 1) use as a vaccine to activate dendritic cells (DCs) and T cells, 2) employing monoclonal antibodies to improve specificity and reduce damage, 3) employing immune adjuvants or complement activators to create a new therapeutic modality, photoimmunotherapy (PIT), and 4) using tumor cells treated with PDT to promote DC production of vaccines.

Increasingly, researchers have combined the immune-activating effects of PDT with immunotherapies that enhance tumor immunogenicity (e.g., immunoadjuvants) or immunotherapies that reduce immunosuppression in the tumor microenvironment (e.g., immune checkpoint inhibitors, anti-PD-1, anti-PD-L1, and anti-CTLA4 antibodies, etc.), which ultimately increased tumor infiltration of cytotoxic CD4^+^/CD8^+^ T cells and memory T cells that produced significant antitumor effects, as shown in **Figure 6**. Hisataka et al. prepared a monoclonal antibody-conjugated photosensitizer capable of targeting epidermal growth factor receptors (EGFRs) with deeper tissue penetration and specific targeting, resulting in effective tumor eradication. When combined with tumor immune checkpoint inhibitors (PD-1/PD-L1), PDT induces regression of photo-irradiated primary and unirradiated distant tumors by inducing a strong tumor-specific immune response (80). Then, some researchers demonstrated that combining PDT with immune adjuvants or complement activators significantly improved PDT efficacy (81). Korbelik et al. found that Mycobacterium cell wall extract (MCWE) could be used as a non-specific immune activator in combination with PDT, which significantly increased immune cell activity. In addition, they found that local application of the complement activator yeast polysaccharide to tumors or systemic application of streptokinase followed by combined PDT could enhance the anticancer effect of PDT and reduce tumor recurrence (82). In addition, Gollnick et al. showed that the lysis products after PDT treatment were able to activate dendritic cells (DCs) and T cells to express IL-12, which enhanced the host anti-tumor immune response (83). Mladen et al. demonstrated that PDT-treated head and neck squamous cell carcinoma cells could be used for vaccination of their loaded mice, and that treatment of the cells with calreticulin prior to injection after which the antitumor effect was significantly increased (84). This shows that PDT combined with immunotherapy has the potential to effectively eradicate the target tumor as well as any residual cancer cells and metastases, triggering the immune memory to prevent tumor recurrence and providing the possibility of a cure.

### 5.5 Photodynamic therapy combined with targeted therapy strategies

Targeting strategies to improve the delivery of photosensitizers to tumor tissues, which can simultaneously enhance the selectivity and efficacy of PDT, have subsequently received widespread attention. There are two main types of targeting strategies: passive targeting and active targeting. A summary of these two strategies is provided below.

**1. Photodynamic combined passive targeted therapy strategy**

**Figure 7:**
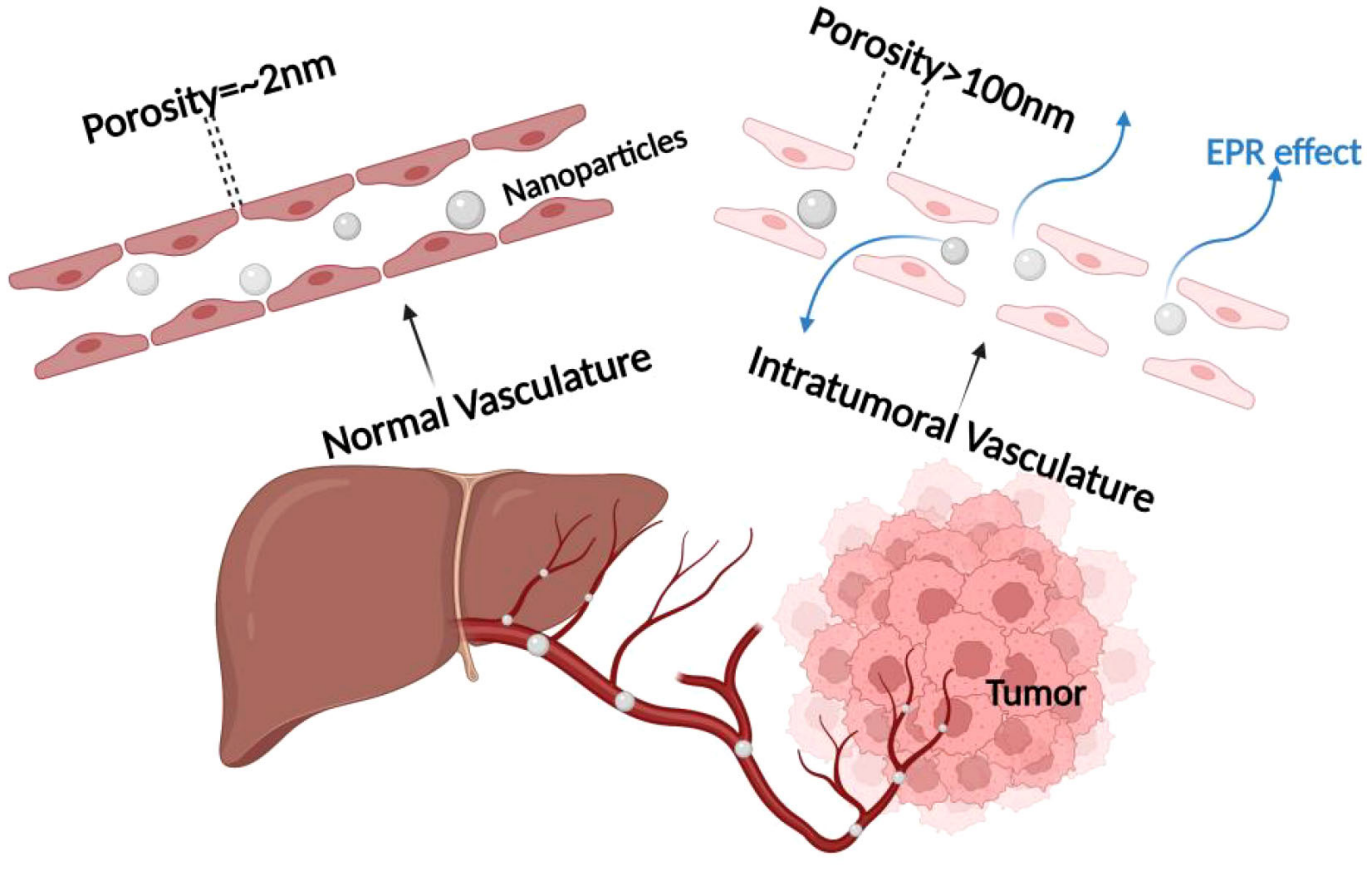
Mechanism of EPR effect. The enhanced permeability and retention (EPR) effect mechanism, which is crucial for passive targeting of nanocarriers in cancer treatment, emphasizing the role of tumor blood vessel fenestrations in facilitating nanoparticle entry into the tumor microenvironment.

Passive targeting is achieved by nanotechnologically modifying photosensitizers into multifunctional nanoparticles and then modulating the size and surface chemistry of the nanoparticle (or macromolecular) drug to promote its selective accumulation within the tumor through enhanced permeability and retention effects (EPR effects). The EPR effects are usually attributed to the rapid growth of cancer cells, which consume local nutrients at a very high rate and induce the rapid generation of structurally incomplete blood vessels, and the leaky pores in these new vessels enhance the penetration of circulating nanoparticles into the tumor microenvironment, whereas penetration in non-malignant tissues is limited by the intact vascular barrier. In addition, nanoparticles tend to be selectively retained in the tumor tissue due to the impaired lymphatic drainage system therein. To achieve passive targeting effects, nanoparticle sizes of 10–200 nm are usually required. In addition to size, other intrinsic properties of the nanoparticles (e.g., shape, charge, hydrophilicity, and blood circulation time) affect the efficiency of passive tumor targeting. Despite some drawbacks of this approach, the effectiveness of EPR-based *in vivo* targeting has been demonstrated in preclinical tumor models. In some early tumor tissues, the role of EPR may be limited by their small size and more regular vascular system. In addition, the irregularity of the vascular space is usually heterogeneous within the tumor mass, resulting in EPR-mediated targeting that is heterogeneous throughout (85–87), as shown in **Figure 7**.

**2. Photodynamic combined active targeted therapy strategy**

**Figure 8:**
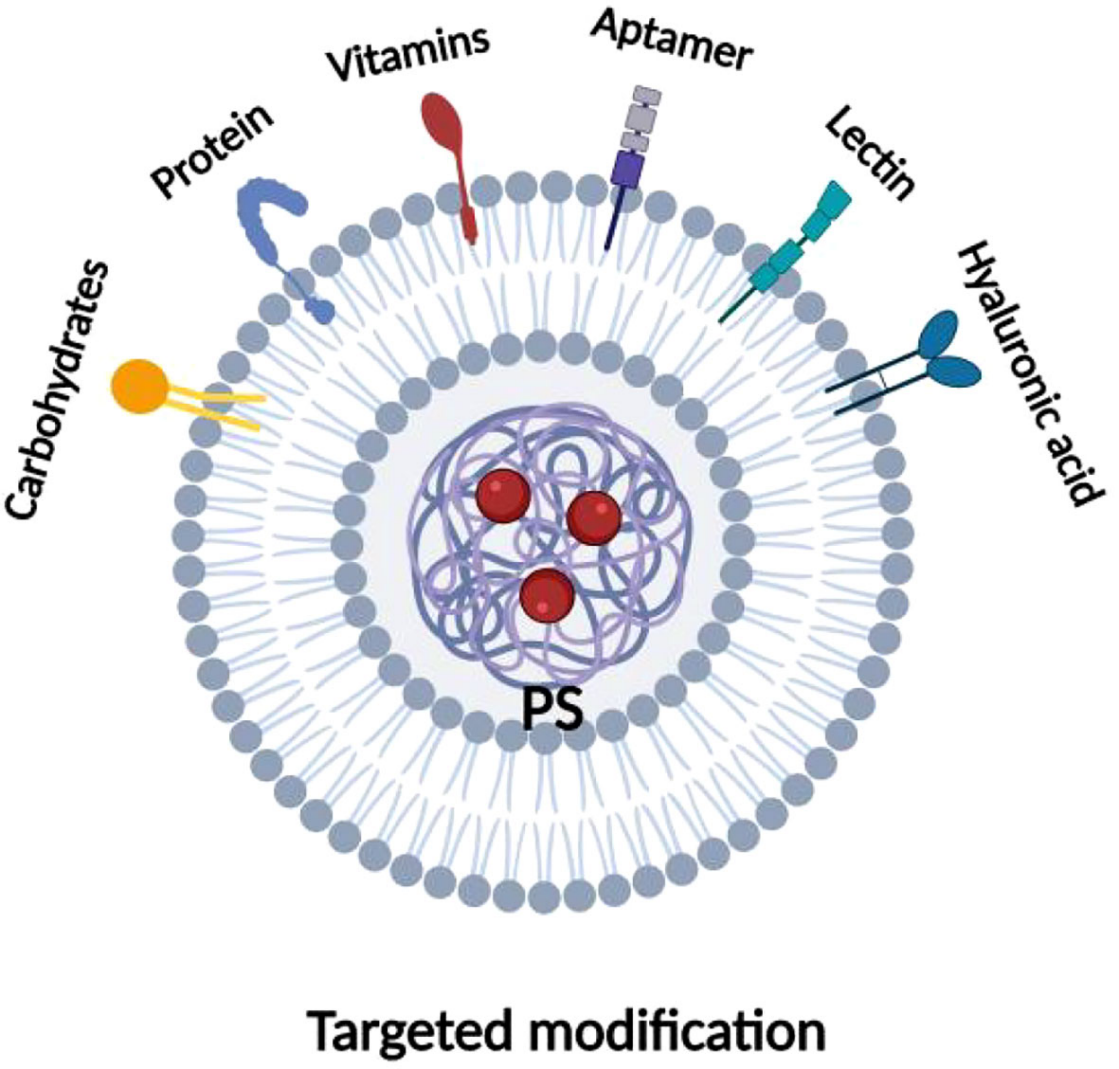
Types of ligand for photosensitizer targeted modifications. Various targeted ligands utilized for modifications of photosensitizers, including proteins, aptamers, hyaluronic acid, lectins, vitamins, lactose, etc.

Active targeting to improve tumor selectivity usually involves modifying the surface of the photosensitizer with several high-affinity ligands that selectively interact with specific receptors overexpressed on the surface of tumor cells, increasing their internalization. These targeted ligands are broadly categorized into peptides (e.g., arginine-glycine-aspartate peptides and epidermal growth factor), proteins (e.g., transferrin and antibodies), aptamers, vitamins (e.g., folic acid and biotin), and carbohydrates, among others, as shown in **Figure 8**. More and more researchers are now applying biological ligands to target photosensitizers with excellent results. The most frequently reported biologic ligands used to target PDT are antibodies. Russell et al. used an anti-HER-2 antibody to modify photosensitizer C11Pc to prepare novel nanomedicines (Anti-HER2-C11Pc-PEG-AuNPs) for PDT combination therapy of breast cancer. In this case, the selective interaction between the anti-HER-2 antibody and the HER-2 receptor on the surface of the tumor cells led to an increased internalization of the drug, thus enhancing its efficacy for PDT. The researchers found that the HER-2 receptor is overexpressed in 10-34% of invasive breast cancers, making it an important target for selective PDT. Additionally, it was found that nanomedicines were effectively internalized, initially located around the cell membrane, and flowed over time to acidic organelles (i.e., lysosomes), ultimately leading to effective cell death (88). Another study also found that peptides can be applied to enhance the active targeting of photosensitizers. Cheng et al. modified EGF peptides on the surface of the photosensitizer Pc4 and coupled them to the surface of gold nanoparticles (AuNPs) to prepare functional AuNPs with the ability to target PDTs, and found that the EGF peptides interacted specifically with EGFR, which is overexpressed on the surface of brain tumor cells, to increase intracellular Pc4 uptake tenfold. Because of the longer interaction between AuNPs and the cell membrane, there is the potential for higher uptake, resulting in more Pc4 being released into the cell via receptor-mediated endocytosis. In contrast to Pc4 from nontargeted AuNPs toward mitochondria, endocytosed Pc4 moves toward endosomes upon internalization. Therefore, targeting brain tumors with EGF peptides facilitates the development of AuNPs that can cross the blood-brain barrier and lead to effective photodynamic tumor cell killing. The fact that the folate receptor is overexpressed in a variety of tumors, including ovarian, breast, and lung cancers, and is expressed at a low level in normal tissues, has made it an important target for cancer therapy (89). Taking advantage of this property, Nair et al. modified folic acid on the surface of the photosensitizer PpIX (90), and Roghayeh et al. also coated PEGylated folic acid on the surface of the photosensitizer dihydroporphyrinol e6 (Ce6) (91), and the results of both experiments showed enhanced internalization of the photosensitizers in cancer cells as compared to epithelial cells and confirmed the specificity of the interaction between folic acid and folate receptors. Due to the elevated dose of intracellular photosensitizer, which in turn enhanced its PDT efficacy. In addition, studies have shown that CD44 glycoprotein is highly expressed in many tumor tissues and is involved in tumor growth, invasion, and metastasis, with specific binding properties to hyaluronic acid. Tham et al. bound a layer of hyaluronic acid to the surface of the photosensitizer zinc phthalocyanine (ZnPc-Si), and selective interactions between the hyaluronic acid and CD44 on the surface of the tumor cells increased ZnPc-Si cellular internalization and enhanced its PDT efficacy. Internalization and enhance its PDT effect, which ultimately effectively inhibited tumor growth, invasion, and metastasis (92). It can be seen that the specific selection of photosensitizers on tumor tissues can be enhanced by PDT combined with a targeted therapy strategy, which increases their internalization enhanced the efficacy of PDT. At the same time, it reduces nonspecific tissue damage and has good potential for clinical application.

## 6 Discussion and outlook

Surgery, chemotherapy, radiotherapy, targeted therapy, and immunotherapy, as basic cancer treatments, have been widely used to treat various tumors and achieved good results, but there are still limitations, such as the high toxicity and low tolerance of chemotherapy drugs, the obvious radioactive damage caused by radiotherapy, and the limited efficacy of targeted and immunotherapy monotherapy, etc., which all limit the efficacy of cancer treatments and reduce the patient’s quality of life (93). Therefore, we need to find a new treatment method to alleviate these problems. Photodynamic therapy (PDT), as a non-invasive tumor treatment, is more targeted and causes less damage to the surrounding normal tissues than other methods. The main effects of PDT are direct toxicity to tumor cells, damage to tumor blood vessels, and anti-tumor immunity 3]. In this paper, we summarize the research on the mechanism of action of PDT in recent years as well as the strategy of its combination therapy, which can achieve better efficacy by combining PDT with other conventional treatment modalities. PDT combined with chemotherapy can reduce the dosage of chemotherapeutic drugs, overcome the MDR of the tumor cells, and improve the survival rate and the quality of life of cancer patients. PDT combined with radiotherapy can increase the sensitivity of the tumors to radiotherapy PDT combined with radiotherapy can increase tumor sensitivity to radiotherapy, reduce radiation dose, alleviate radiotherapeutic injury, and significantly improve patients’ quality of life; PDT combined with anti-tumor immunotherapy can effectively inhibit the growth and drug resistance of local primary tumors, and at the same time, fight against distant metastasis of tumors and prevent their recurrence. The process of immune activation by PDT may be related to the PDT therapeutic dose, the intensity of inflammatory response, the release of antigens of the responsive target cells of PDT, and positive regulation of the immune cells. Moreover, PDT combined with targeted therapy can effectively improve photosensitizer internalization, enhance PDT tumor specificity, and obtain a better therapeutic index through two different therapeutic strategies: passive targeting and active targeting.

With the continuation of the production of light sources with increased power by biomedical optics technologies, as well as the capability for the use of multiple fibers and decreased size and costs, the expectation is for the interest in phototherapies to remain high. In fact, phototherapies are currently being developed for a wide variety of applications (1). In parallel with Artificial Intelligence (AI) advancements, the development of novel photosensitizers is another promising area in cancer treatment. The innovation in photosensitizer design aims to improve their selectivity, efficacy, and safety, thereby expanding the therapeutic potential of PDT. The synergy between AI-driven treatment planning and advanced photosensitizers could lead to more personalized and effective cancer therapies. By integrating AI technologies into radiotherapy, healthcare providers can optimize treatment planning, enhance patient care, and potentially improve the overall success rates of cancer treatments (94).

Although better anti-tumor effects have been observed with PDT combined with other therapeutic strategies, there are still some limitations of PDT: 1) There are fewer basic experimental and clinical studies on PDT combination therapy, which cannot provide reliable evidence for clinical application. We need more animal studies and clinical trials to make up for the lack of safe and effective research data in this field.2) We need more basic studies to comprehensively elucidate: the mechanism by which the PDT combination therapy strategy improves the tumor therapeutic effect.3) The problem of the weak laser penetration of PDT is still not solved, which restricts the scope of application of the combination therapy, and we should carry out more studies to find the most effective We should conduct more research to find the most effective photosensitizer and light source technology or explore new treatment modes. In conclusion, PDT in combination with other therapies can effectively improve its antitumor effects, and at the same time provide survival benefits and improve the quality of life of patients by alleviating the toxic side effects of treatment. As mentioned earlier, the combination of PDT with radiotherapy, chemotherapy, targeted therapy, and immunotherapy provides new ideas and opportunities for tumor treatment.
